# Chromatin remodeler CHD8 is required for spermatogonial proliferation and early meiotic progression

**DOI:** 10.1093/nar/gkad1256

**Published:** 2024-01-16

**Authors:** Kenta Nitahara, Atsuki Kawamura, Yuka Kitamura, Kiyoko Kato, Satoshi H Namekawa, Masaaki Nishiyama

**Affiliations:** Department of Histology and Cellular Biology, Graduate School of Medical Sciences, Kanazawa University, Kanazawa 920-8640, Japan; Department of Gynecology and Obstetrics, Graduate School of Medical Sciences, Kyushu University, Fukuoka 812-8582, Japan; Department of Histology and Cellular Biology, Graduate School of Medical Sciences, Kanazawa University, Kanazawa 920-8640, Japan; Department of Microbiology and Molecular Genetics, University of California, Davis, Davis, CA 95616, USA; Department of Gynecology and Obstetrics, Graduate School of Medical Sciences, Kyushu University, Fukuoka 812-8582, Japan; Department of Microbiology and Molecular Genetics, University of California, Davis, Davis, CA 95616, USA; Department of Histology and Cellular Biology, Graduate School of Medical Sciences, Kanazawa University, Kanazawa 920-8640, Japan

## Abstract

Meiosis is a key step during germ cell differentiation, accompanied by the activation of thousands of genes through germline-specific chromatin reorganization. The chromatin remodeling mechanisms underpinning early meiotic stages remain poorly understood. Here we focus on the function of one of the major autism genes, CHD8, in spermatogenesis, based on the epidemiological association between autism and low fertility rates. Specific ablation of *Chd8* in germ cells results in gradual depletion of undifferentiated spermatogonia and the failure of meiotic double-strand break (DSB) formation, leading to meiotic prophase I arrest and cell death. Transcriptional analyses demonstrate that CHD8 is required for extensive activation of spermatogenic genes in spermatogonia, necessary for spermatogonial proliferation and meiosis. CHD8 directly binds and regulates genes crucial for meiosis, including H3K4me3 histone methyltransferase genes, meiotic cohesin genes, HORMA domain-containing genes, synaptonemal complex genes, and DNA damage response genes. We infer that CHD8 contributes to meiotic DSB formation and subsequent meiotic progression through combined regulation of these meiosis-related genes. Our study uncovers an essential role of CHD8 in the proliferation of undifferentiated spermatogonia and the successful progression of meiotic prophase I.

## Graphical Abstract

**Graphical Abstract:**
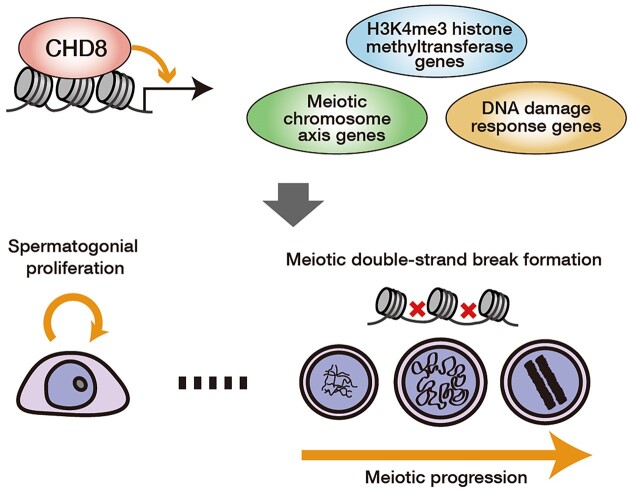

## Introduction

Male infertility has recently become a major health problem, accounting for approximately 50% of total infertility (1,2). One of the major difficulties in deciphering the pathology of infertility is that we must understand a germ cell-specific differentiation process, namely meiosis. During the transition from mitosis to meiosis, chromatin undergoes structural reorganization, and its remodeling causes transcriptional activation of crucial spermatogenic genes (3–6). However, the mechanisms directing chromatin remodeling in meiosis remain poorly understood.

Several major chromatin remodeling factors, including SWI/SNF (7,8), ISWI (9) and INO80 (10,11), are required for the relatively late stage of meiotic prophase I (especially during and after the pachytene stage). Recent studies revealed that HELLS, a member of SWI/SNF chromatin remodeler, works with a histone methyltransferase PRDM9 to regulate programmed DNA double-strand breaks (DSBs) and its mutation results in differentiation arrest at the mid-pachytene stage (7,8). Further, another major chromatin remodeler—INO80—cooperates with polycomb group complex 2 and is required for DSB repair and meiotic progression after the pachytene stage (10,11). In addition, in fission yeast, Hrp1 and Hrp3, members of the chromodomain helicase DNA-binding protein (CHD) chromatin remodeling family, regulate transcription programs to alter chromatin structure and facilitate meiotic recombination (12). These lines of evidence highlight that progressive chromatin remodeling is required to generate haploid sperm.

The chromatin remodeler chromodomain helicase DNA-binding protein 8 (CHD8) is primarily known as an autism gene because it is frequently found mutated in individuals with autism spectrum disorder (ASD) (13–16). On the other hand, CHD8 has been reported to play an essential role in the development of various non-neural organs, such as hematopoietic stem cells (17,18), adipocytes (19), and intestinal cells (20). In this study, we sought to determine the function of CHD8 in spermatogenesis, in part because of the epidemiological association between intellectual disabilities including autism, and low fertility (21,22). In particular, reduced fertility rate is more substantial in male patients with ASD (22). To date, a relationship between ASD and low fertility remains to be elucidated, however. Therefore, revealing the role of CHD8 in spermatogenesis is of great importance.

We examined the function of CHD8 during spermatogenesis by establishing a germ-cell specific *Chd8* loss-of-function mouse model. We observed that *Chd8* ablation in germ cells leads to testicular atrophy and infertility. Upon closer inspection, CHD8-deficient germ cells exhibited two independent phenotypes: a gradual depletion of undifferentiated spermatogonia and acute differentiation arrest before the pachytene stage during meiotic prophase I. Transcriptional analyses demonstrated that CHD8 is required for extensive activation of spermatogenic genes in spermatogonia. We confirmed that CHD8 is associated with the activation of genes required for meiosis, namely H3K4me3 histone methyltransferase genes, meiotic cohesin genes, HORMA domain-containing genes, synaptonemal complex genes, and DNA damage response (DDR) genes. Our results uncover a crucial function of CHD8, regulating histone methyltransferases, meiotic chromosome axes, and DDR for the successful progression of meiosis prophase I.

## Materials and methods

### Mice

The generation of *Chd8*^F/–^ and *Chd8*^F/F^ mice was described previously (23). *Chd8*-floxed mice were generated by loxP sites flanking exons 11 to 13. Offspring were backcrossed onto the C57BL/6J line for at least nine generations. *Ddx4-Cre* and *Ddx4-CreER*^T2^ transgenic mice were described previously (24,25). *Ddx4-Cre*; *Chd8*^+/F^ male mice were crossed with *Chd8*^F/F^ female mice to produce *Ddx4-Cre*; *Chd8*^F/–^ male mice, and *Ddx4-CreER*^T2^; *Chd8*^+/F^ mice were crossed with *Chd8*^F/F^ mice to produce *Ddx4-CreER*^T2^; *Chd8*^F/F^ male mice. In all experiments, unless otherwise mentioned, *Ddx4-Cre*; *Chd8*^F/–^ mice were compared with *Ddx4-Cre*; *Chd8*^+/F^ littermates, and *Ddx4-CreER*^T2^; *Chd8*^F/F^ mice were compared with *Ddx4-CreER*^T2^; *Chd8*^+/F^ littermates.

Mice were genotyped by PCR analysis of genomic DNA with primers for *Chd8* (5′-CCCAAAAGACCAAATCAAACAAAC-3′, 5′-CCATAG GCTGAAGAACCGTAATTG-3′, and 5′-AGGCTT AGAAACCCGTCGAG-3′) and *Cre* (5′-AGGTTCGTTCACT CATGGA-3′ and 5′-TCGACCAGTTTAGTTACCC-3′) (26). All animals were maintained under specific pathogen-free conditions, and all experiments were performed under the approval of the Animal Care Committee of Kanazawa University (protocol no. KINDAI 6-2135).

### Tamoxifen treatment

To inducibly delete *Chd8* in adult male mice, 8-week-old *Ddx4-CreER*^T2^; *Chd8*^F/F^ male mice, as well as their littermate controls, were treated with tamoxifen. In short, tamoxifen (Sigma-Aldrich) was dissolved in corn oil (Sigma-Aldrich) to a final concentration of 20 mg/ml. Each mouse was intraperitoneally injected at a dose of 3.0 mg for five consecutive days. The day of the final injection was counted as 0 day post-tamoxifen (dpt).

### Fertility assessment

The reproductive ability of *Ddx4-Cre*; *Chd8*^F/–^ male mice in comparison with *Ddx4-Cre*; *Chd8*^+/F^ male littermates was measured as previously described (27). In brief, 8-week-old male mice were mated with two or three wild-type females of the same age up to 4 weeks. 16 days after the visual confirmation of vaginal plug, pregnant mice were sacrificed and litter sizes were measured.

### Sperm count

The evaluation of sperm number was performed as previously described (28). In short, a pair of epididymides of 8-week-old *Ddx4-Cre*; *Chd8*^F/–^ mice and their littermate controls were dissected and minced in HTF medium (FUJIFILM Irvine Scientific) for 20 min at 37°C. The fluid was collected near the peripherals. A total sperm count was assessed in the final suspension using a hemacytometer. For images, the sperm suspension was air-dried and the smear was stained with hematoxylin.

### Antibodies

A rat monoclonal antibody and a rabbit polyclonal antibody to CHD8 were produced as described previously and used for chromatin immunoprecipitation (ChIP) and immunoblot, respectively (23). Other antibodies used for immunofluorescence, immunoblot, ChIP, and MACS analyses included those to TRA98 (ab82527, Abcam), PLZF (Santa Cruz Biotechnology sc-28319 for immunofluorescence of cryosections, Abcam ab189849 for whole-mount immunostaining), GFRA1 (AF560, R&D Systems), Ki67 (550609, BD Biosciences), STRA8 (ab49602, Abcam), SYCP1 (ab15090, Abcam), SYCP3 (ab15093, Abcam), γH2AX (05-636, Millipore), DMC1 (sc-373862, Santa Cruz Biotechnology), HSP90 (610419, BD Biosciences), CD90.2/Thy1 (130-121-278, Miltenyi Biotec), CD117/c-Kit (130-091-224, Miltenyi Biotec) and H3K4me3 (ab8580, Abcam).

### Histology and immunohistochemistry

For hematoxylin-eosin staining, mouse testes were fixed overnight at 4°C with Bouin's solution. After embedding in paraffin, the sections (thickness of 4 μm) were deparaffinized and stained with hematoxylin and eosin.

For cryosectioning, mouse testes were fixed with 4% paraformaldehyde (PFA) for 4 hours. After dehydration overnight with 30% sucrose at 4°C, the samples were embedded in Tissue-Tek O.C.T. compound (Sakura Finetek) and frozen at −80°C. The cryosections (thickness of 7 μm) were rehydrated in PBS. The immunostaining samples for PLZF were autoclaved in sodium citrate buffer (10 mM sodium citrate, 0.05% Tween 20, pH 6.0) for 10 min at 121°C for antigen retrieval.

To immunostain single-cell-suspended samples, suspended cells were fixed with 1% PFA on Poly-L-Lysine-coated slides for 30 min. After washing with PBS, the slides were incubated with antibodies.

Whole-mount immunostaining was performed as previously described (29,30). In brief, after removal of tunica albuginea in PBS, seminiferous tubules were incubated with Dulbecco's modified Eagle's medium (DMEM) supplemented with 1 mg/ml collagenase (Wako) and 0.1 mg/ml DNase I (Wako) for 10 minutes at 37°C. The samples were then incubated with 4% PFA for 4 hour at 4°C. After washing with PBS, the tubules were treated with a graded methanol series (25, 50, 75, 95% and twice in 100% MeOH) for 10 min at room temperature, followed by permeabilization with a 4:1:1 mixed solution of MeOH/DMSO/H2O2 for 30 min at room temperature and rehydration with 50% MeOH, 25% MeOH and twice in PBS for 10 min each at room temperature.

Chromosome spread analysis was performed as previously described with minor modifications (31,32). Mouse testes were collected and detached from tunica albuginea in PBS. The seminiferous tubules were extracted in hypotonic buffer (30 mM Tris [pH 7.5], 17 mM trisodium citrate dihydrate and 50 mM sucrose). The cells were suspended in 100 mM sucrose and placed on slides in fixative buffer (1% PFA and 0.1% Triton X-100).

TUNEL assay was performed using the MEBSTAIN Apoptosis TUNEL Kit Direct (MBL 84445), according to the manufacturer's instructions.

Immunostaining samples, including the cryosections, single-cell-suspended samples, whole-mount samples, and chromosome spreads, were exposed to 3% BSA and 0.1% Triton X-100 before incubation overnight at 4°C with primary antibodies. After washing with PBS, the samples were incubated with Alexa Fluor 488– or Alexa Fluor 546–conjugated goat secondary antibodies (Thermo Fisher Scientific) for one hour at room temperature. The samples were counterstained by DAPI (Wako) and mounted with Fluoromount (Diagnostic BioSystems).

### Images

Images of hematoxylin-eosin staining were captured with All-in-One Microscope BZ-X800 (Keyence). The immunofluorescence and TUNEL assay images were captured with a laser-scanning confocal microscope (LSM700, Zeiss) and processed with ZEN imaging software (Zeiss) and ImageJ software (NIH). γH2AX signal intensity was measured by quantifying nuclear area after correcting the background signal using Fiji software (33).

### Immunoblot analysis

Total protein was extracted with a lysis buffer (50 mM Tris–HCl (pH 7.5), 150 mM NaCl, 0.5% Triton X-100), supplemented with protease inhibitor cocktail (Wako). The extracts were subjected to immunoblot analysis as previously described (19). Quantification of CHD8 protein abundance was performed by comparing it to the protein abundance of HSP90 (loading control) using Fiji software (33).

### Enrichment of spermatogonia populations

Isolation of spermatogonia was described previously (34,35). In summary, testes were collected in a dish containing DMEM. After detachment of tunica albuginea, the testes were digested with 1 mg/ml collagenase (Wako) for 20 min at 37°C to remove interstitial cells. The tubules were washed with DMEM and digested with 2.5 mg/ml trypsin and 0.1 mg/ml DNase I (Wako) for 20 min at 37°C. The reaction was stopped by adding DMEM supplemented with 10% fetal bovine serum. Cells were filtered with a 40-μm strainer to remove Sertoli cells, and the cell suspension was placed in a 24-well plate for 30 min to promote adhesion and precipitation of the remaining somatic cells. Cells in the supernatant were washed with MACS buffer (PBS supplemented with 0.5% BSA and 5 mM EDTA) and incubated with CD117/c-Kit MicroBeads (Miltenyi Biotec) for 15 min on ice. Cells were washed and resuspended with MACS buffer. After filtering with a 40-μm strainer, c-Kit^+^ cells were isolated using MS columns (Miltenyi Biotec). Cells in the flowthrough fraction were washed and incubated with CD90.2/Thy1 MicroBeads (Miltenyi Biotec) for 15 min on ice. Cells were washed and resuspended with MACS buffer. After filtering with a 40-μm strainer, Thy1^+^ cells were isolated using MS columns (Miltenyi Biotec). The purity of MACS-purified spermatogonia was confirmed by immunostaining, reverse transcription quantitative polymerase chain reaction (RT-qPCR), and RNA-seq analysis.

### RT-qPCR analysis and RNA-seq analysis

Thy1^+^ and c-Kit^+^ spermatogonia were isolated from the testes of three male pups at 10 dpp for each analysis. Total RNA was extracted from the enriched spermatogonia or the cells in the flow-through with the use of a PureLink RNA Mini Kit (Thermo Fisher Scientific).

For RT-qPCR analysis, the extracted RNA was subjected to reverse transcription with ReverTra Ace RT mix with gDNA remover (Toyobo). After synthesizing cDNA, real-time PCR analysis was conducted using Luna Universal qPCR Master Mix (New England Biolabs) in a Thermal Cycler Dice Real Time System III (Takara Bio). The quantitative data was normalized by the abundance of *Gapdh* mRNA. The PCR primers (sense and antisense, respectively) were as follows: *Gapdh*, 5′-GCCTGGAGAAACCTGCCAAGTATG-3′ and 5′-GAGTGGGAGTTGCTGTTGAAGTCG-3′; *Plzf*, 5′-CTGGGACTTTGTGCGATGTG-3′ and 5′- CGG TGGAAGAGGATCTCAAACA-3′; *Stra8*, 5′-ACAACCTA AGGAAGGCAGTTTAC-3′ and 5′-GACCTCC TCTAAGCTGTTGGG-3′.

For RNA-seq analysis, the preparation of a cDNA library and sequencing with the use of a NovaSeq 6000 system (Illumina) was conducted by Rhelixa Co., Ltd (Tokyo, Japan). The paired-end reads were trimmed with Trimmomatic (version 0.39), mapped against the mouse (mm10) genome with the use of HISAT2 (version 2.1.0), and counted with featureCounts (version 2.0.1).

Differential gene expression analysis was performed using edgeR (version 3.16) (Dataset S1) (36). We employed a quantile-adjusted conditional maximum likelihood method to estimate common and tagwise dispersions. An exact test for a single comparison was used to assess differential expression. The differential expression of genes with an absolute log_2_(fold-change) > 0.5 and false discovery rate (FDR) < 0.05 were considered significant.

GSEA was performed as previously described with GSEA software (version 4.3.2) (37). The gene sets for ‘SSC self-renewal ∼ initiation of differentiation’, ‘spermatogonial differentiation ∼ meiotic entry’, and ‘meiosis prophase I’ (Figure 5D, E, Dataset S2) were obtained from Hermann *et al.* (38) and ‘Cell cycle process’ and ‘E2F targets’ were obtained from the Molecular Signature Database (37). The groups of representative genes (Figure 5F, G) were obtained from Soh et al. (39) and Hermann et al (38). Gene ontology analysis was performed using DAVID web tools for differentially expressed genes. The gene ontology analysis of up-regulated genes in Thy1^+^ spermatogonia was omitted because no significant enrichment was confirmed due to the small number of up-regulated genes (20 genes).

### ChIP-seq analysis

ChIP-seq was performed essentially as described previously (23). For CHD8 ChIP-seq, we used Thy1^+^ and c-Kit^+^ spermatogonia from three male mice of *Ddx4-Cre*; *Chd8*^+/F^ and *Ddx4-Cre*; *Chd8*^F/–^ at 10 dpp or unsorted testicular cells from three wild-type male mice at 12 dpp. For H3K4me3 ChIP-seq, we used c-Kit^+^ spermatogonia from three male mice of *Ddx4-Cre*; *Chd8*^+/F^ and *Ddx4-Cre*; *Chd8*^F/–^ at 10 dpp. Two biological replicates and three biological replicates were generated for each spermatogonial sample and unsorted testicular sample, respectively. The extracted DNA was size-selected with AMPure XP (Thermo Fisher Scientific) and an E-gel electrophoresis system (Thermo Fisher Scientific). We prepared a DNA library with a ThruPLEX DNA-Seq kit (Takara Bio). The library was sequenced with the use of a NovaSeq 6000 system (Illumina). The paired-end reads were trimmed with Trimmomatic (version 0.39) and mapped against the mouse (mm10) genome with the use of bowtie2 (version 2.4.5), and duplicated reads were removed with SAMtools (version 1.14).

For the identification of significant peaks, ChIP data was compared with matched input control using MACS2 peak caller (version 2.2.7.1, with the option ‘-p 1e-5–gsize mm–nomodel–extsize 160’). Peak annotation was performed with ChIPseeker (version 1.34.1). Overlaps of ChIP peaks and significantly down-regulated genes were intersected with bedtools (version 2.30.0). Average aggregate profiles and heatmaps were created using deepTools (version 3.5.1) (40). Briefly, bigwig coverage files were created using the ‘bamCompare’ tool with normalization using a method of signal extraction scaling (41). The files were converted to the average aggregate profiles and heatmaps using the ‘computeMatrix’ tool with maxThreshold 1000 and ‘plotHeatmap’ tool. The enrichment of chromatin occupancy at specific loci was visualized using the IGV genome browser (version 2.13.1). The locations of PRDM9-dependent and PRDM9-independent DSB hotspot centers were determined as previously described using SPO11-oligos (42) and PRDM9 ChIP-seq analysis (43).

The distribution probabilities of the overlapping gene lists were calculated using the R package phyper. The Pearson correlation between biological replicates of CHD8 ChIP-seq profiles (bin = 10 kb) in Thy1^+^ and c-Kit^+^ spermatogonia and box plot analysis of CHD8 binding amount in fragments per kilobase of transcript per million mapped reads (FPKM) was generated using SeqMonk (Babraham Institute, version 1.48.1).

### Statistical analyses

Quantitative data are shown as mean ± SEM. Statistical analysis by the unpaired two-tailed Student's *t* test, Welch's *t* test, and the Mann–Whitney *U* test was performed with Microsoft Excel software. Data were assumed to be normally distributed, otherwise the Mann–Whitney *U* test was applied. *P* value and FDR of <0.05 were considered statistically significant. Although no statistical power analysis was performed beforehand, our sample sizes were in accordance with those generally adopted in the field (31,32,34,35,44).

## Results

### CHD8 is highly expressed in spermatogonia and is indispensable for spermatogenesis

**Figure 1.**
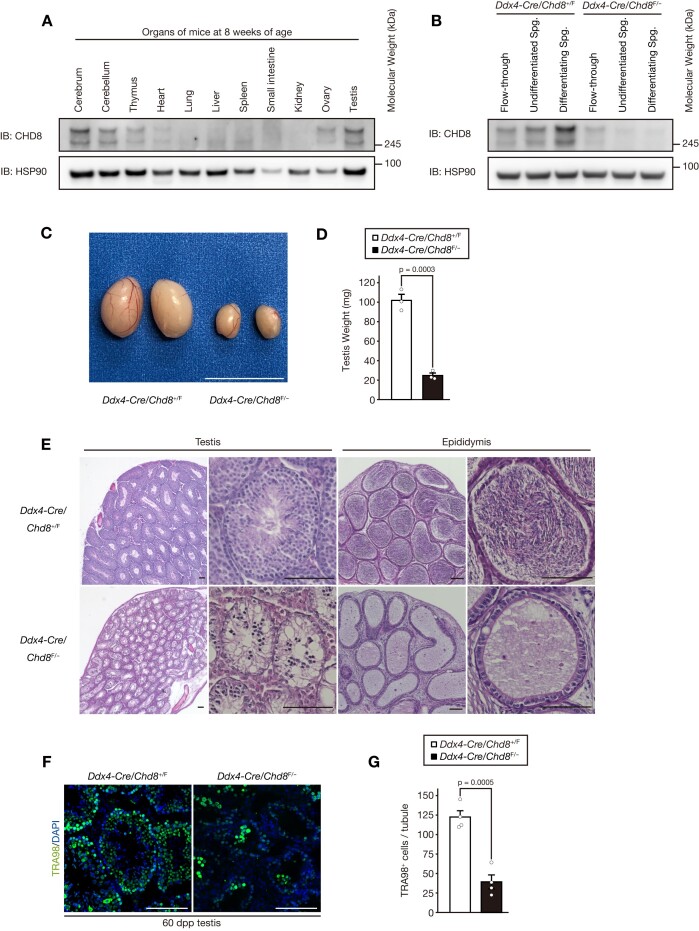
CHD8 is highly expressed in spermatogonia and is indispensable for spermatogenesis. (**A**) Immunoblot analysis of CHD8 and HSP90 (loading control) was performed for the indicated organs of wild-type mice at 8 weeks of age. (**B**) Immunoblot of CHD8 and HSP90 (loading control) was performed for flow-through, undifferentiated spermatogonia, and differentiated spermatogonia from the mice of the indicated genotypes at 8 weeks of age. (**C**) Appearance of testes collected from the mice of the indicated genotypes at 8 weeks of age. Scale bar, 1 cm. (**D**) Testicular weight of the mice of the indicated genotypes (*n* = 3 per genotype) at 8 weeks of age. (**E**) Hematoxylin-eosin staining of the testes and epididymides from the mice of the indicated genotypes at 8 weeks of age. Scale bars, 100 μm. (**F**) Immunofluorescence staining of TRA98 in the testes from the mice of the indicated genotypes at 8 weeks of age. Scale bar, 100 μm. (**G**) Quantification of the number of cells positive for TRA98 per tubule in images as in (F) (*n* = 4 per genotype). All data are means ± SEM. *P* values are calculated using the two-tailed Student's *t* test. Spg., spermatogonia.

To understand which tissues CHD8 is expressed in, we first compared the levels of CHD8 protein in multiple organs. Unsurprisingly, as one of the major autism-causing proteins, CHD8 was highly expressed in the nervous system, in the cerebrum and cerebellum. It was also expressed in the testes (Figure 1A). Additional immunoblot analysis revealed that CHD8 was particularly enriched in Thy1^+^ undifferentiated spermatogonia and c-Kit^+^ differentiating spermatogonia among testicular subpopulations (Figure 1B). This implies that CHD8 is likely to have a distinct function in the spermatogonial population in testes.

Since global deletion of *Chd8* results in embryonic lethality (45), we adopted a conditional knockout mouse model in which *Chd8* alleles are floxed and removed by Cre recombinase (23). By crossing male *Ddx4-Cre*; *Chd8*^+/F^ mice with *Chd8*^F/F^ females, we generated *Ddx4-Cre*; *Chd8*^F/–^ male mice to achieve *Chd8* ablation in the germline from embryonic day 15.5 (Supplementary Figure S1A) (25). Immunoblot verified that CHD8 was efficiently deleted in mutant spermatogonial cells (Figure 1B). Adult mice with germline-specific *Chd8* deletion displayed apparent atrophy of testes and infertility (Figure 1C and D, Supplementary Figure S1B). Histological analysis confirmed a severe defect in spermatogenesis and the loss of post-meiotic spermatids or sperm in the testes and epididymides of adult *Ddx4-Cre*; *Chd8*^F/–^ mice (Figure 1E). Immunofluorescence analyses of *Chd8*-ablated testicular sections verified a decrease in the number of germ cells (i.e. TRA98^+^ cells) (Figure 1F and G). These findings suggest that CHD8 protein is enriched in spermatogonia and that CHD8 is essential for spermatogenesis.

### CHD8 regulates the proliferation of spermatogonial stem cells (SCCs)

**Figure 2.**
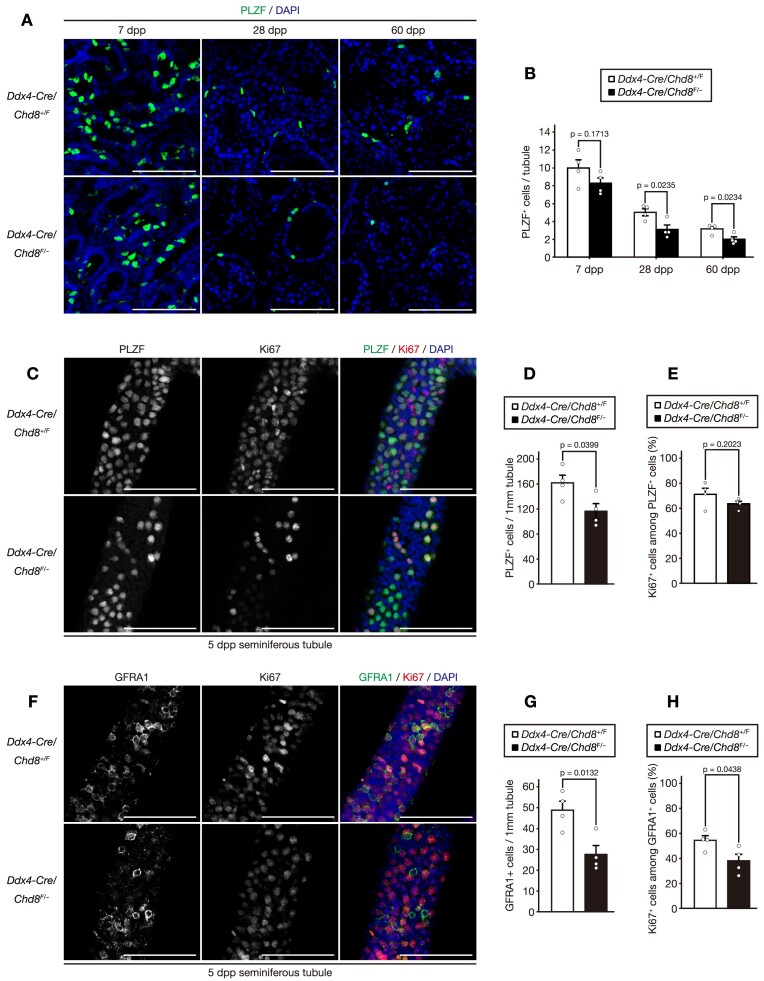
CHD8 regulates proliferation of spermatogonial stem cells (SCCs). (**A**) Immunofluorescence staining of PLZF in the testes from the mice of the indicated genotypes at 7, 28 and 60 dpp. Scale bars, 100 μm. (**B**) Quantification of the number of the cells positive for PLZF per tubule in images as in (A) (*n* = 4 per genotype). (**C**) Whole-mount immunofluorescence staining of PLZF and Ki67 in the seminiferous tubule from the mice of the indicated genotypes at 5 dpp. Scale bars, 100 μm. (**D**) Quantification of the number of the cells positive for PLZF per 1mm tubule length in images as in (C) (*n* = 4 per genotype). (**E**) Quantification of the number of the cells positive for Ki67 among PLZF + cells in the seminiferous tubules in images as in (C) (*n* = 4 per genotype). (**F**) Whole-mount immunofluorescence staining of GFRA1 and Ki67 in the seminiferous tubule from the mice of the indicated genotypes at 5 dpp. Scale bars, 100 μm. (**G**) Quantification of the number of cells positive for GFRA1 per 1mm tubule length in images as in (F) (*n* = 4 per genotype). (**H**) Quantification of the number of the cells positive for Ki67 among GFRA + cells in the seminiferous tubules in images as in (F) (*n* = 4 per genotype). All data are means ± SEM. *P* values are calculated using the two-tailed Student's *t* test. Dpp, days post-partum.

Based on the expression profile of enriched CHD8 protein in spermatogonia, we initially investigated the role of CHD8 in the SSC pool. From 7 to 60 days post-partum (dpp), the number of PLZF^+^ undifferentiated spermatogonia progressively decreased in *Chd8* ablated testes relative to controls (Figure 2A and B).

To examine this phenotype in detail, we performed a whole-mount immunofluorescence analysis at 5 dpp to evaluate undifferentiated spermatogonia prior to initial spermatogonial differentiation caused by the first wave of spermatogenesis. The number of PLZF^+^ cells was, indeed, significantly reduced in the *Chd8* mutant tubules (Figure 2C and D). Cells positive for GFRA1 (a marker for cells with stem-cell characteristics within undifferentiated spermatogonia) (30,46) were also decreased in number in CHD8-deficient tubules (Figure 2F and G). We also analyzed the proliferative capability of undifferentiated spermatogonia by staining them for the active cell cycle marker Ki67. Although the proportion of PLZF^+^ cells in the cell cycle (Ki67^+^) did not differ between the CHD8-deficient tubules and the controls (Figure 2E), fewer *Chd8* mutant GFRA1^+^ cells were actively dividing, as measured by Ki67 positivity (Figure 2H). We confirmed that the proportion of GFRA1^+^/PLZF^+^ cells was comparable between the two groups (Supplementary Figure S2A and B). These findings suggest that CHD8’s function is associated with active cell division of undifferentiated spermatogonia. Taken together, *Chd8* ablation results in gradual depletion of SSC cell number due to a reduction in the number of cells that are actively proliferating.

### Loss of CHD8 leads to arrest before pachytene of meiotic prophase I and subsequent cell death

**Figure 3.**
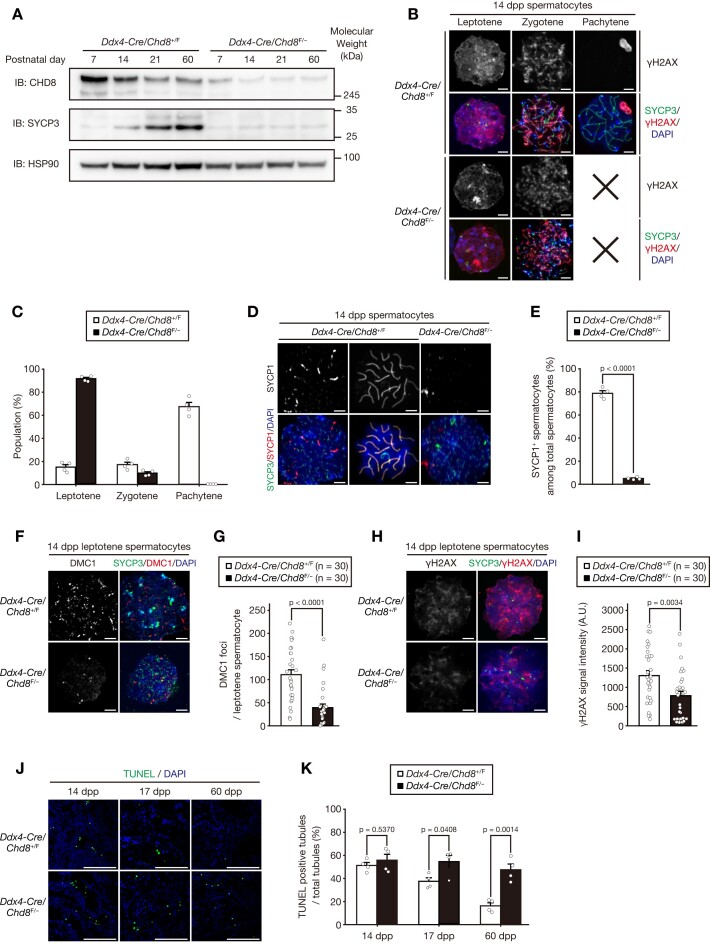
Loss of CHD8 leads to arrest before pachytene of meiotic prophase I and subsequent cell death. (**A**) Immunoblot analysis of CHD8, SYCP3 and HSP90 (loading control) was performed for the testes from the mice of the indicated genotypes at 7, 14, 21 and 60 dpp. (**B**) Immunofluorescence staining of SYCP3 and γH2AX in the spermatocytes from the mice of the indicated genotypes at 14 dpp. × denotes no cell entry in the indicated category. Scale bars, 5μm. (**C**) Quantification of the number of cells in each indicated meiotic phase as in (B) (*n* = 4 per genotype). (**D**) Immunofluorescence staining of SYCP3 and SYCP1 in the spermatocytes from the mice of the indicated genotypes at 14 dpp. Scale bars, 5 μm. (**E**) Quantification of the number of the cells positive for SYCP1 among total spermatocytes in images as in (D) (*n* = 4 per genotype). (**F**) Immunofluorescence staining of SYCP3 and DMC1 in leptotene spermatocytes from the mice of the indicated genotypes at 14 dpp. Scale bars, 5 μm. (**G**) Quantification of the number of DMC1^+^ foci per leptotene spermatocyte in images as in (F) (*n* = 30 per genotype). (**H**) Immunofluorescence staining of SYCP3 and γH2AX in leptotene spermatocytes from the mice of the indicated genotypes at 14 dpp. Scale bars, 5 μm. (**I**) Quantification of γH2AX signal intensity in images as in (H) (*n* = 30 per genotype). (**J**) TUNEL assay in the testes from the mice of the indicated genotypes at 14, 17 and 60 dpp. Scale bars, 100 μm. (**K**) Quantification of the number of tubules containing TUNEL positive cells among total tubules in images as in (J) (n = 4 per genotype). All data are means ± SEM. *P* values are calculated using the two-tailed Student's t test (C, E and K) or the Mann–Whitney *U* test (G and I). Dpp, days post-partum. A.U., arbitrary unit.

We next analyzed the role of CHD8 during SSC differentiation. We first measured the number of cells that were positive for STRA8, a marker of differentiating spermatogonia and preleptotene spermatocytes (47). The number of STRA8^+^ cells was comparable between *Chd8*-ablated testes and the controls (Supplementary Figure S3A and B). Immunoblot analysis, however, revealed that throughout development, CHD8-deficient testes had low levels of SYCP3, a major component of meiotic chromosome axes (32,48), while a greater enrichment of SYCP3 was observed in the controls from 14 dpp onwards (Figure 3A). These results indicate that *Chd8* ablation resulted in the depletion of germ cells expressing SYCP3.

To elucidate the role of CHD8 in meiotic progression, we performed a chromosome spread analysis at 14 dpp, when most spermatocytes in the first wave of murine spermatogenesis reach the pachytene stage of meiotic prophase I (32,49). While the majority of control spermatocytes advanced to a pachytene stage, most *Chd8*-ablated spermatocytes were arrested at the leptotene stage, and none reached the pachytene stage (Figure 3B and C). This meiotic arrest during meiotic prophase I was also confirmed in CHD8-deficient spermatocytes of adult mice (Supplementary Figure S3C and D). This acute differentiation arrest was confirmed by a decrease in the number of SYCP1 (a marker for the central element of synaptonemal complex (32,48)) positive cells in *Chd8*-mutant spermatocytes (Figure 3D and E), indicating that CHD8 is essential for synapsis formation and subsequent progression in meiotic prophase I. Moreover, the γH2AX signal intensity and the number of DMC1 foci were reduced in CHD8-deficient spermatocytes at the leptotene stage (Figure 3F–I), suggesting that CHD8 deficiency causes the failure of meiotic DSB formation and subsequent homologous recombination.

Next, we performed a TUNEL staining analysis to trace the fate of *Chd8*-ablated spermatocytes after arrest in meiotic prophase I. Although the number of TUNEL-positive cells was similar at 14 dpp between the control and mutant testes, TUNEL positivity was significantly higher in *Chd8*-ablated testes compared to control testes at 17 and 60 dpp (Figure 3J and K). This significant increase of cell death at 17 dpp, the stage at which the second wave of spermatogenesis has not initiated meiosis (44,50), implies that CHD8-deficient first-wave spermatocytes underwent cell death in the middle of meiotic prophase I, especially at the mid-pachytene stage. Collectively, CHD8-deficient germ cells fail to differentiate at an early stage of meiotic prophase I, followed by consequent cell death (Supplementary Figure S3E).

### CHD8 is essential for the maintenance of adult spermatogenesis through two distinct functions

**Figure 4.**
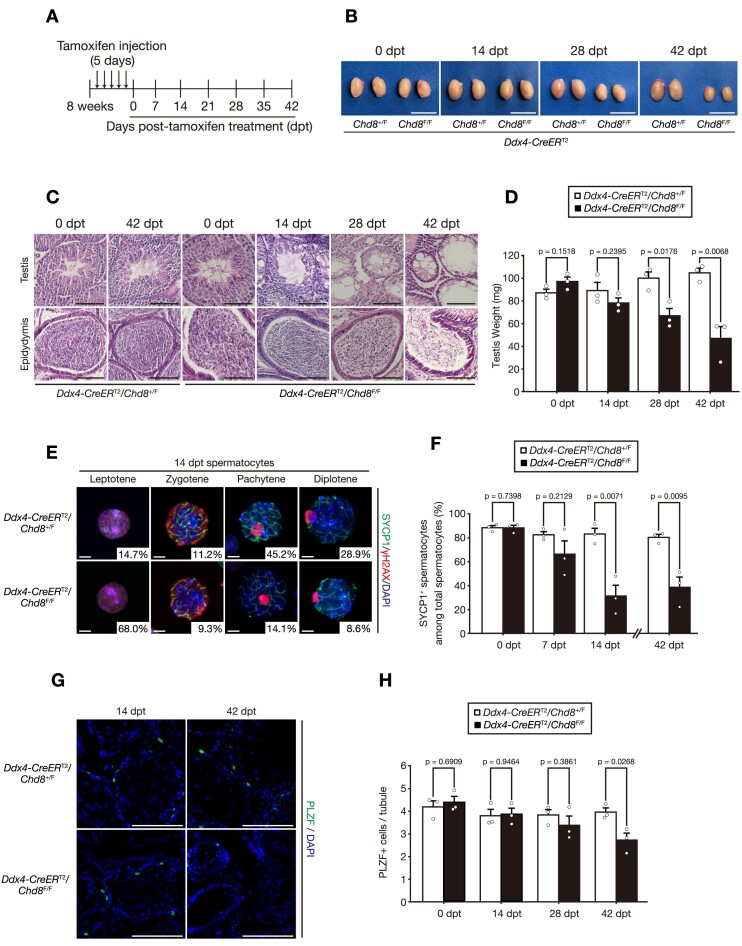
CHD8 is essential for the maintenance of adult spermatogenesis through two distinct functions. (**A**) Schematic strategy of inducible *Chd8* deletion by tamoxifen injection to mice at 8 weeks of age for 5 consecutive days. (**B**) Appearance of the testes collected from the mice of the indicated genotypes at each indicated date after tamoxifen treatment. Scale bar, 1 cm. (**C**) Hematoxylin–eosin staining of the testes and epididymides of mice with the indicated genotypes at each indicated date after tamoxifen treatment. Scale bars, 100 μm. (**D**) Testicular weight in mice with the indicated genotypes (*n* = 3 per genotype) at each indicated date after tamoxifen treatment. (**E**) Immunofluorescence staining of SYCP3 and γH2AX in spermatocytes from mice of the indicated genotypes at 14 dpt. The numbers represent the mean proportion at each meiotic phase among total spermatocytes (*n* = 3 per genotype). Scale bars, 5 μm. (**F**) Quantification of the number of the cells positive for SYCP1 among total spermatocytes in images at each indicated date after tamoxifen treatment (*n* = 3 per genotype). (**G**) Immunofluorescence staining of PLZF in the testes from the mice of the indicated genotypes at each indicated date after tamoxifen treatment. Scale bars, 100 μm. (**H**) Quantification of the number of the cells positive for PLZF per tubule in images as in (G) (*n* = 3 per genotype). All data are means ± SEM. *P* values are calculated using the two-tailed Student's *t* test. Dpt, days post-tamoxifen.

Since male reproductive organs continuously generate mature gametes from the SSC pool, we proceeded to examine the role of CHD8 in adult spermatogenesis. For this purpose, we used tamoxifen-inducible Cre recombinase under the control of the *Ddx4* promoter (i.e. *Ddx4-CreER*^T2^) (24). After treatment of 8-week-old male mice with tamoxifen (Figure 4A), acquired deletion of *Chd8* in germ cells (*Ddx4-CreER*^T2^; *Chd8*^F/F^) resulted in gradual testicular atrophy and a defect in sperm production (Figure 4B–D). We confirmed that the CHD8 protein level significantly declined from 7 dpt and thereafter (Supplementary Figure S4A and B). In a detailed evaluation, we initially observed an acute depletion of spermatocytes after the zygotene stage of meiotic prophase I as early as 14 dpt and thereafter (Figure 4E and F). The number of mature sperm in an epididymis started to decrease at 28 dpt (Supplementary Figure S4C and D). Given that germ cells require approximately 20 days to become mature sperm after completion of meiosis (50,51), the loss of mature sperm at 28 dpt can be viewed as a direct result of the meiotic arrest.

The number of *Chd8*-inducibly-deleted undifferentiated spermatogonia (PLZF^+^ cells), on the other hand, remained comparable with the control until 28 dpt, followed by a significant decrease at 42 dpt (Figure 4G and H). These findings (gradual loss of undifferentiated spermatogonia occurring after the acute depletion of meiotic cells after pachynema in CHD8-defient germ cells) indicate that CHD8 has two independent critical functions in two different differentiation phases, namely undifferentiated spermatogonia and meiotic spermatocytes. Collectively, CHD8 plays two crucial but independent roles in the maintenance of adult spermatogenesis, preserving the SSC population and safeguarding the progression of meiotic prophase I.

### CHD8 is associated with the extensive activation of spermatogenic genes, including stem cell, spermatogonial differentiation, and meiosis-related genes

**Figure 5.**
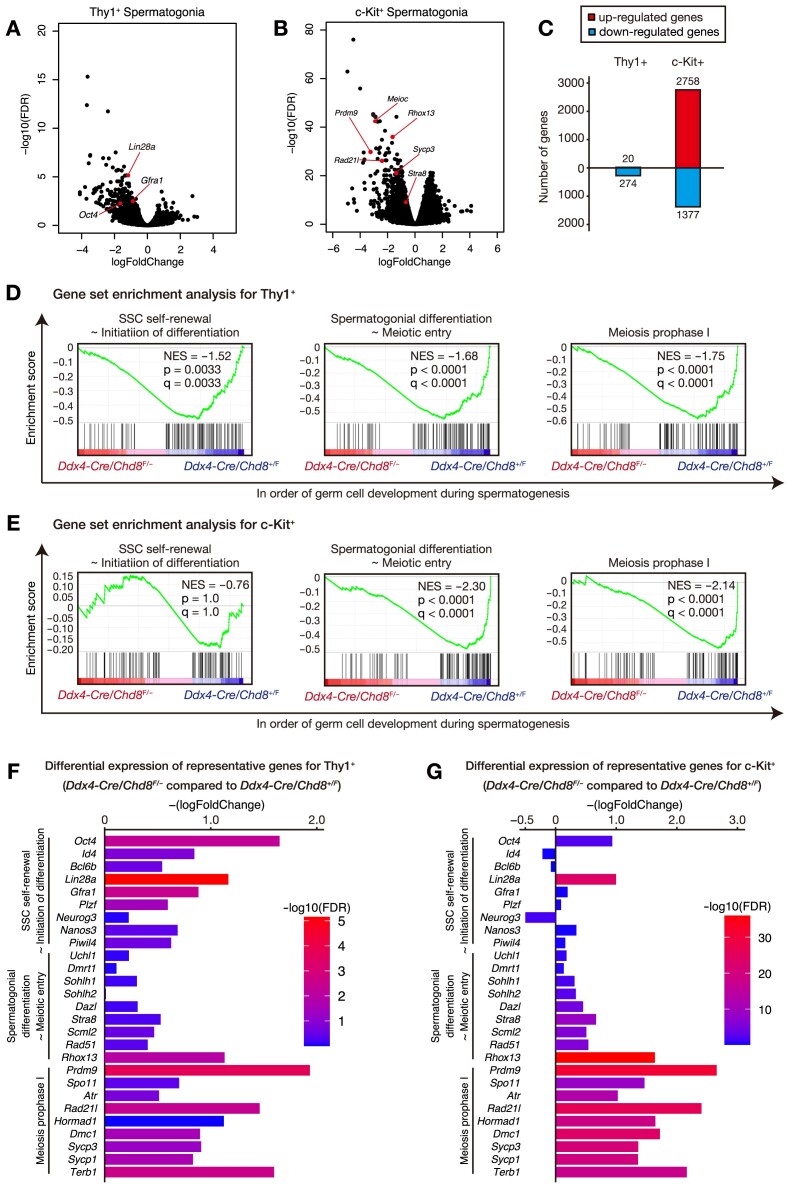
CHD8 is associated with the extensive activation of spermatogenic genes, including stem cell, spermatogonial differentiation, and meiosis-related genes. (A and B) Volcano plots for differentially expressed genes in Thy1^+^ (**A**) and c-Kit^+^ (**B**) spermatogonia of *Ddx4-Cre*; *Chd8*^F/–^ mice compared with *Ddx4-Cre*; *Chd8*^+/F^ mice at 10 dpp (*n* = 3 per genotype). (**C**) The number of differentially expressed genes in Thy1^+^ and c-Kit^+^ spermatogonia. (D and E) GSEA plot of gene expression change among genes specific to three spermatogenic phases in Thy1^+^ (**D**) and c-Kit^+^ (**E**) spermatogonia. (**F** and **G**) Gene expression change of representative genes in three spermatogenic phases in Thy1^+^ (F) and c-Kit^+^ (G) spermatogonia of *Ddx4-Cre*; *Chd8*^F/–^ mice compared with *Ddx4-Cre*; *Chd8*^+/F^. Differentially expressed genes were defined as genes with an absolute log_2_(fold-change) > 0.5 and FDR < 0.05. FDR, false discovery rate. Dpp, days post-partum. NES, net enrichment score.

These findings (protein enrichment of CHD8 in spermatogonia, gradual exhaustion of *Chd8*-mutant undifferentiated spermatogonia, and acute arrest of *Chd8*-mutant spermatocytes at an early phase in meiotic prophase I) imply that the crucial function of CHD8 in spermatogenesis is in spermatogonia. To analyze gene expression changes in spermatogonia after *Chd8* depletion, we performed RNA sequencing (RNA-seq) for Thy1^+^ undifferentiated spermatogonia and c-Kit^+^ spermatogonia purified from testicular cells at 10 dpp using magnetic-activated cell sorting (MACS). The purity of Thy1^+^ undifferentiated spermatogonia and c-Kit^+^ spermatogonia purified by MACS was confirmed by immunostaining, RT-qPCR analysis, and RNA-seq analysis (Supplementary Figure S5A–G), and found to be largely consistent with high purity as reported in the previous studies (34,35). Our RNA-seq analysis demonstrated that *Chd8* ablation resulted in extensive gene expression changes in spermatogonia, while the change was more profound in c-Kit^+^ cells (Figure 5A and B). We observed that 20 and 2758 genes were up-regulated in *Chd8* mutant Thy1^+^ and c-Kit^+^ spermatogonia, respectively (Figure 5C). Gene ontology analysis of these up-regulated genes in c-Kit^+^ spermatogonia showed spermatogenesis-unrelated gene categories, including cell adhesion and angiogenesis, possibly reflecting the function of CHD8 in suppressing the expression of non-lineage-specific genes (Supplementary Figure S5H). On the other hand, we confirmed down-regulation of 274 genes in Thy1^+^ and 1377 genes in c-Kit^+^ CHD8-deficient cells (Figure 5C). Notably, gene ontology analyses of these down-regulated genes in both Thy1^+^ and c-Kit^+^ cells showed terms associated with meiosis, while ‘stem cell maintenance’ was only associated with Thy1^+^ cells. Terms associated with the DDR, such as ‘Cellular response to DNA damage stimulus’ and ‘DNA repair’, were only present in c-Kit^+^ cells (Supplementary Figure S5I and J). These findings suggest that CHD8 selectively activates the transcriptional programs necessary for early-stage spermatogenesis at each spermatogonial stage.

To understand the gene expression patterns of the individual stages of spermatogenesis, we performed a gene set enrichment analysis (GSEA) for genes selectively expressed in the three stages of early-stage spermatogenesis, namely ‘SSC self-renewal ∼ initiation of differentiation’, ‘spermatogonial differentiation ∼ meiotic entry’, and ‘meiosis prophase I’ (38). The GSEA revealed that transcriptional activities in all three categories were widely down-regulated in *Chd8* deficient spermatogonia, except for the initial phase of the ‘SSC self-renewal ∼ initiation of differentiation’ phase in c-Kit^+^ cells (Figure 5D and E), possibly due to the low expression level of SSC-related genes in CHD8 control c-Kit+ cells (Supplementary Figure S5G), without much room for further down-regulation. We also confirmed significant down-regulation of ‘Cell Cycle Process’ and ‘E2F targets’ in *Chd8*-mutant Thy1^+^ cells (Supplementary Figure S5K and L), consistent with our previous observation that *Chd8* ablation results in the impairment of cell-cycle activities in undifferentiated spermatogonia.

To analyze the data in detail, we then examined the expression of individual representative genes in early-stage spermatogenesis. While most selected genes were down-regulated by *Chd8* ablation in Thy1^+^ and c-Kit^+^ spermatogonia, steep down-regulation was observed among stem cell-related genes, such as *Oct4* and *Lin28a*, as well as extensive down-regulation in meiosis-related genes, such as *Prdm9* and *Rad21l* (Figure 5F and G). Taken together, the loss of CHD8 in germ cells resulted in extensive down-regulation of spermatogonial genes, especially genes related to stem cells and meiosis.

### CHD8 transcriptionally regulates H3K4me3 histone methyltransferase, meiotic chromosome axis proteins, and DDR proteins to safeguard meiotic progression

**Figure 6.**
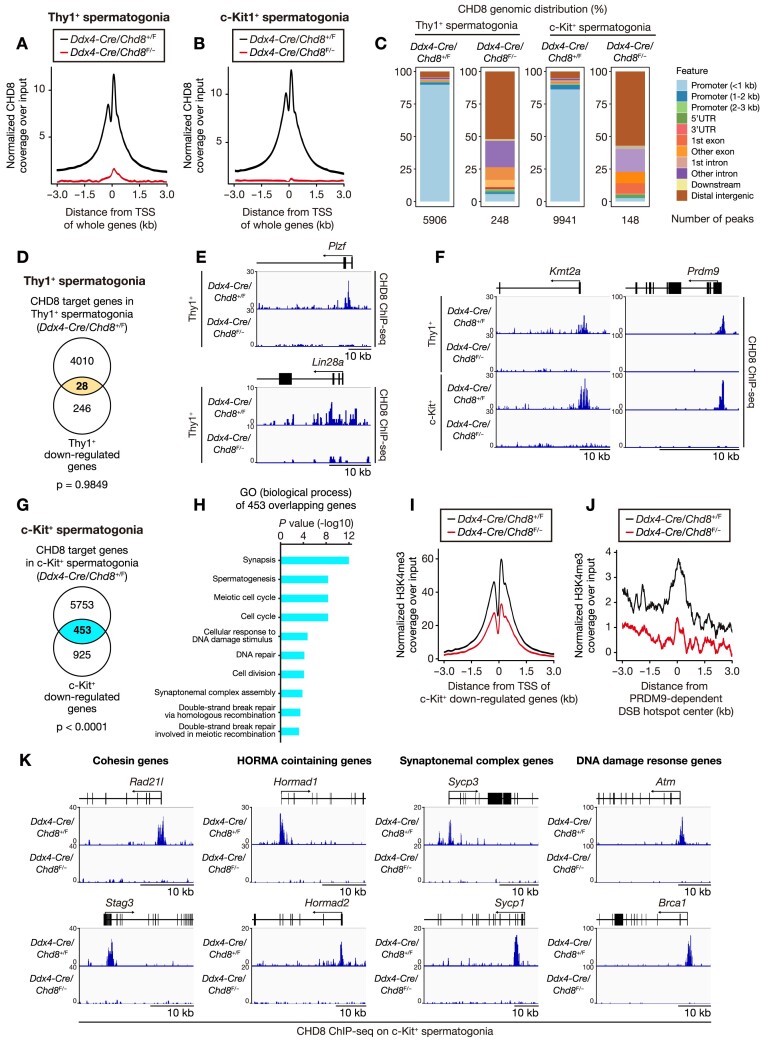
CHD8 transcriptionally regulates H3K4me3 histone methyltransferase, meiotic chromosome axis proteins, and DDR protein responses to safeguard meiotic progression. (A and B) Signal density of CHD8 peaks relative to TSSs of whole genes determined by ChIP-seq analyses of (**A**) Thy1^+^ and (**B**) c-Kit^+^ spermatogonia from the mice of the indicated genotypes. (**C**) Composition of CHD8 binding peaks determined by ChIP-seq analyses. (**D**) Venn diagram showing overlap between CHD8 target genes (4038 genes) determined by CHD8 ChIP-seq analysis in Thy1^+^ spermatogonia of *Ddx4-Cre*; *Chd8*^+/F^ and genes whose expression is down-regulated in Thy1^+^ spermatogonia (274 genes) of *Ddx4-Cre*; *Chd8*^F/–^ mice compared with *Ddx4-Cre*; *Chd8*^+/F^ mice. (**E**) CHD8 ChIP-seq data at *Plzf* and *Lin28a* genes in Thy1^+^ spermatogonia from the mice of the indicated genotypes, viewed in the Integrative Genomic Viewer browser. (**F**) CHD8 ChIP-seq data at histone methyltransferase genes in Thy1^+^ and c-Kit^+^ spermatogonia from the mice of the indicated genotypes, viewed in the Integrative Genomic Viewer browser. (**G**) Venn diagram showing overlap between CHD8 target genes (6206 genes) determined by CHD8 ChIP-seq analysis in c-Kit^+^ spermatogonia of *Ddx4-Cre*; *Chd8*^+/F^ and genes whose expression is down-regulated in c-Kit^+^ spermatogonia (1378 genes) of *Ddx4-Cre*; *Chd8*^F/–^ mice compared with *Ddx4-Cre*; *Chd8*^+/F^ mice. (**H**) Gene ontology analysis of the 453 overlapping genes shown in (G). (**I**) Signal density for H3K4me3 ChIP-seq peaks relative to TSSs of genes whose expression was down-regulated in c-Kit^+^ spermatogonia from the mice of the indicated genotypes. (**J**) Signal density for H3K4me3 ChIP-seq peaks relative to PRDM9-dependent DSB hotspot centers in c-Kit^+^ spermatogonia from the mice of the indicated genotypes. (**K**) CHD8 ChIP-seq data for cohesin genes, HORMA domain-containing genes, synaptonemal complex genes, and DDR genes in c-Kit^+^ spermatogonia from mice of the indicated genotypes viewed in the Integrative Genomic Viewer browser. Dpp, days post-partum. DSB, double-strand break. *P* values are calculated using the ‘phyper’ function in R.

To obtain mechanistic insight into the relationship between CHD8 and spermatogonial gene expression, we performed ChIP-seq analysis of CHD8 in Thy1^+^ and c-Kit^+^ spermatogonia at 10 dpp with antibodies to CHD8. A substantial fraction of the CHD8 binding sites resided within ± 1 kb of the transcription start sites (TSSs) in *Ddx4-Cre*; *Chd8*^+/F^ spermatogonia, whereas CHD8 peaks were barely detected in *Ddx4-Cre*; *Chd8*^F/–^ spermatogonia (Figure 6A–C, Supplementary Figure S6A). The reproducibility of the CHD8 ChIP-seq analysis was confirmed by two independent biological replicates (Supplementary Figure S7A, B).

To determine how CHD8 regulates genes involved in SCC maintenance and meiotic progression, we analyzed the ChIP-seq results using differentially expressed genes detected in the RNA-seq (both Thy1^+^ and c-Kit^+^ cells). Of 4038 genes to whose TSSs CHD8 bound in Thy1^+^ spermatogonia, we found that 28 genes were down-regulated in Thy1^+^ spermatogonia in the RNA-seq (Figure 6D, Dataset S3). Although the number of overlapping genes of Thy1^+^ was proportionally small, most of these overlapping genes are common to the CHD8-bound genes in all three independent ChIP-seq analyses (i.e. CHD8 ChIP-seq on Thy1^+^ spermatogonia, c-Kit^+^ spermatogonia, and unsorted testicular cells) (Supplementary Figure S6B), suggesting that the overlap was not caused by mere coincidence. We confirmed that the overlapping 28 genes contain *Plzf* and *Lin28a* (Figure 6E), required for SSC maintenance and proliferation (52,53), as well as H3K4me3 histone methyltransferase genes (Figure 6F). Moreover, in Thy1^+^ ChIP-seq, we observed that CHD8 binds to the TSSs of E2F target genes (Supplementary Figure S6C, D). CHD8 particularly binds to the TSSs of *Atad2* and *Smc4*, two E2F targets in the overlapping 28 genes (54,55) (Supplementary Figure S6E).

Of 6206 genes whose TSSs CHD8 bound to in c-Kit^+^ spermatogonia, we found that 453 genes were down-regulated in c-Kit^+^ spermatogonia in the RNA-seq (Figure 6G, Dataset S3). Within the 6206 CHD8 bound genes, CHD8 binding enrichment was similar when comparing the 5753 non-dysregulated genes and 453 down-regulated ones. Gene ontology analysis of the 453 overlapping genes revealed meiosis-related terms, including meiotic cell cycle, synaptonemal complex, and DSB repair as well as DDR (Figure 6H), whereas gene ontology analysis of the 5753 CHD8-bound but non-dysregulated genes showed only spermatogenesis-unrelated terms (Supplementary Figure S6G). This indicates that CHD8 binds to the TSSs of both cell type-nonspecific genes and cell type-specific genes, but its mutation causes down-regulation only in the cell type-specific genes.

In further analysis of the 453 overlapping genes of c-Kit^+^, we identified H3K4me3 histone methyltransferase genes, *Kmt2a* and *Prdm9* (Figure 6F), responsible for creating histone H3 lysine4 trimethylation (H3K4me3) near TSSs to activate gene transcription (56) and selectively depositing H3K4me3 at DSB sites during meiotic recombination (57,58), respectively. We therefore carried out an additional ChIP-seq analysis of H3K4me3 in the c-Kit^+^ spermatogonia of *Ddx4-Cre*; *Chd8*^F/–^ mice and *Ddx4-Cre*; *Chd8*^+/F^ mice at 10 dpp. H3K4me3 ChIP-seq analysis revealed that binding was decreased at the TSSs of significantly down-regulated genes in RNA-seq of c-Kit + spermatogonia (Figure 6I, Supplementary Figure S6H) as well as at DSB hotspots bound by PRDM9 (43) and SPO11 (detected by SPO11-bound oligonucleotides [SPO11-oligos]) (42) (Figure 6J, Supplementary Figure S6I). We observed that H3K4me3 peaks at PRDM9-independent DSB hotspots were also decreased (Supplementary Figure S6J), possibly due to the down-regulation of meiosis-nonspecific H3K4me3 histone methyltransferase activities. Furthermore, the marked reduction of H3K4me3 accumulation was confirmed around TSSs of genes down-regulated in *Chd8*-mutant spermatogonia, including *Lin28a*, *Stra8*, and *Dmc1*, which are crucial for SSC proliferation, spermatogonial differentiation, and meiotic progression, respectively (Supplementary Figure S6K) (51–53,58).

In addition to H3K4me3 histone methyltransferase genes, the 453 overlapping genes of c-Kit + include genes essential for the completion of meiosis prophase I. CHD8 binds to the promoters of meiotic cohesin genes (i.e. *Rad21l* and *Stag3*), HORMA domain-containing genes (i.e. *Hormad1* and *Hormad2*), synaptonemal complex genes (i.e. *Sycp3* and *Sycp1*), and DDR genes (i.e. *Atm* and *Brca1*) (Figure 6K). These meiotic chromosome axis genes and DDR genes cooperate with PRDM9 to contribute to the formation of meiotic DSB formation (59,60), in agreement with the function of CHD8 during meiosis (Figure 3H, I). These results indicate that CHD8 is associated with the activation of genes crucial for SCC proliferation and meiotic progression at each differentiation phase, namely Thy1^+^ and c-Kit^+^ spermatogonia.

## Discussion

Here we show that CHD8 is crucial for maintaining undifferentiating spermatogonia and meiotic progression in spermatogenesis. *Chd8* mutation in germ cells resulted in an acute arrest before the pachytene stage of meiotic prophase I as well as gradual depletion of SCCs. In contrast to other chromatin remodelers that are involved with the relatively late stage of meiotic prophase I (7–11), our study reveals that CHD8 regulates the dynamic early stages of meiotic prophase I through a novel pathway.

In mammalian meiosis, DSBs are marked by PRDM9 in a species-specific manner (61,62). Mutation of *Prdm9* per se does not cause severe defects in meiotic progression in the PWD background (63), suggesting that PRDM9 functions with other proteins at the onset of meiotic prophase. A previous study revealed that the interplay between PRDM9 and STAG3 is required for meiotic DSB formation by recruiting essential DSB-promoting proteins (60). In addition, in fission yeast, the recruitment of a HORMA domain-containing protein to the chromosome axis facilitates meiotic DSB formation and chromosome synapsis (64), and, in mice, RAD21L mediates the loading of HORMAD1 for subsequent synapsis formation (65). Moreover, PRDM9 and the representative DDR gene, ATM, cooperate to regulate SPO11-bound recombination intermediates in meiotic recombination (59). Because the meiotic chromosome axis genes and the *Atm* gene are direct targets of CHD8-dependent regulation, we infer that, through the interplay of these meiosis-related genes, CHD8 contributes to meiotic DSB formation and subsequent meiotic progression. At the same time, because CHD8 regulates the expression of various genes related to spermatogenesis, it is difficult to exclude apart indirect effects of CHD8 in spermatogenesis.

In preceding studies, the CHD family has been shown to play a pivotal role in stem cell function during spermatogenesis (35,66,67). CHD2 maintains spermatogonial self-renewal by stabilizing mRNA of stem cell-related genes (67), whereas CHD4 preserves the SSC pool through interaction with a transcription factor, SALL4, to regulate spermatogonial genes (66). In this study, we demonstrated that CHD8 contributes to the maintenance of the SSC population by regulating proliferative activities. Among stem cell-related genes, *Lin28a* was down-regulated in *Chd8*-ablated Thy1^+^ spermatogonia. Since LIN28A is a key regulator of SSC proliferation (52,53), the gradual decrease in the number of CHD8-deficient undifferentiated spermatogonia compared to the control may be due to a difference in the proliferative activities of SSCs. In addition, CHD8 has been shown to cooperate with E2F to regulate cell proliferation through gene transcription (26,68). Our GSEA results showing down-regulation of E2F target genes and cell-cycle-checkpoint genes in *Chd8* deficient Thy1^+^ spermatogonia reinforce the association between CHD8 and proliferative activity of undifferentiated spermatogonia.

In our ChIP-seq analyses, we observed that CHD8 binds to the TSSs of a relatively large number of genes (4038 genes in Thy1^+^ and 6206 genes in c-Kit^+^ spermatogonia). Most of these CHD8 targets were not down-regulated in our RNA-seq, consistent with a previous report in the context of cerebellar cells (26). As a possible explanation for this result, we speculate that CHD8, as a general chromatin remodeler, broadly binds to cell type-nonspecific genes, and only a small portion of CHD8 peaks is associated with cell type-specific genes. The cell type-nonspecific genes are constitutively transcribed by the collaborative action of many promoters (69,70). Thus their expression could possibly be minimally influenced by the ablation of *Chd8*. The expression of cell type-specific genes including meiosis-related genes, on the other hand, could be more dependent on CHD8 and therefore affected by the ablation of *Chd8*.

Another aspect of this study is that it highlights the relationship between the autism gene *Chd8* and infertility. Although epidemiological studies have shown a correlation between intellectual disabilities (including autism) and low fertility, it was unclear whether the impairment of social function caused by intellectual disability per se lowers the fertility rate or whether other factors are involved (21,22). Our study provides biological information on this relationship between the two diseases. Disorders caused by *Chd8* mutation have a shared molecular etiology involving H3K4me3 histone methyltransferase activity, both in the testes (this study) and the nervous system (71). Based on the common molecular function of CHD8, future therapeutic options for autism could also be applicable as infertility treatments, and vice versa. Overall, our study uncovers an essential role of CHD8 in early meiotic progression by transcriptionally regulating H3K4me3 histone methyltransferases. These findings provide important insight into the association between chromatin remodeling and early meiotic progression as well as biological information on the relationship between infertility and autism.

## Supplementary Material

gkad1256_Supplemental_Files

## Data Availability

The accession number for the RNA-seq and ChIP-seq data presented in this study is GEO: GSE252121.
