# Supplementary material for: Chromatin remodeler CHD8 is required for spermatogonial proliferation and early meiotic progression
Source: Nucleic Acids Res. 2024 Jan 16;52(6):2995–3010. doi: 10.1093/nar/gkad1256 (PMC11014243; doi:10.1093/nar/gkad1256)
Supplement: gkad1256_Supplemental_Files [file gkad1256_supplemental_files.zip › nitaharaNAR2si.pdf]

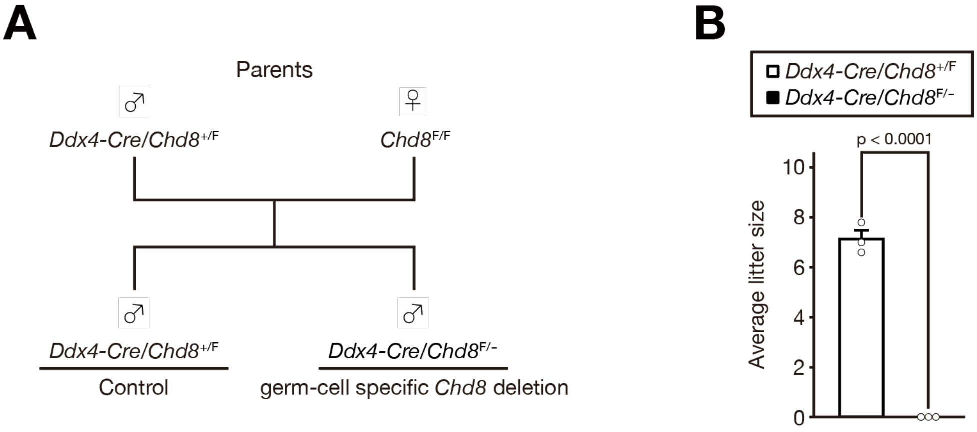

Figure S1. CHD8 is highly expressed in spermatogonia and is indispensable for spermatogenesis

(A) Schematic of the crossing strategy to obtain *Ddx4-Cre*; *Chd8*$^{+/F}$ mice (control) and *Ddx4-Cre*; *Chd8*$^{F/-}$ (germ-cell specific *Chd8* deletion).

(B) Quantification of the average litter size in the mice of the indicated genotypes at 8 weeks of age (n = 3 mice per genotype). Data are means ± SEM. *P* values are calculated using the two-tailed Student's *t* test.

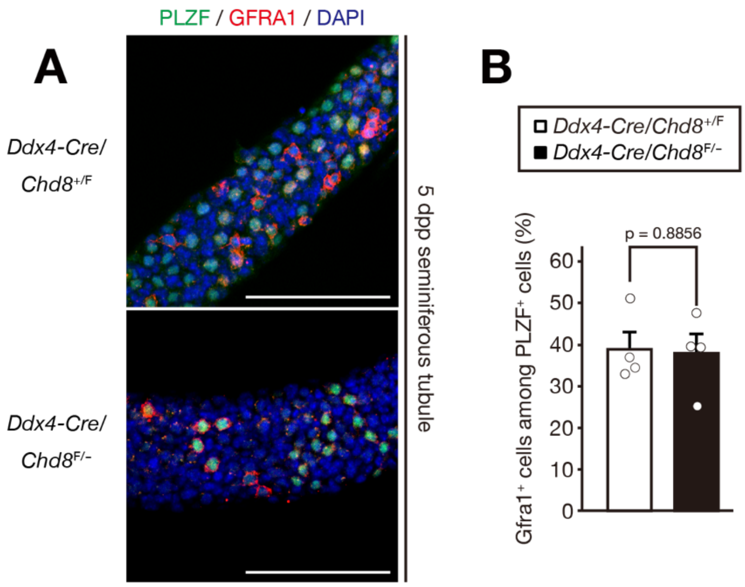

Figure S2. CHD8 regulates the proliferation of spermatogonial stem cells (SCCs)

(A) Whole-mount immunofluorescence staining of PLZF and GFRA1 in seminiferous tubules from mice of the indicated genotypes at 5 dpp. Scale bars, 100μm.

(B) Quantification of the number of the GFRA1^{+} cells among PLZF^{+} cells in the seminiferous tubules in images as in (A) (n = 4 mice per genotype). Data are means ± SEM. *P* values are calculated using the two-tailed Student's *t* test. Dpp, day post-partum.

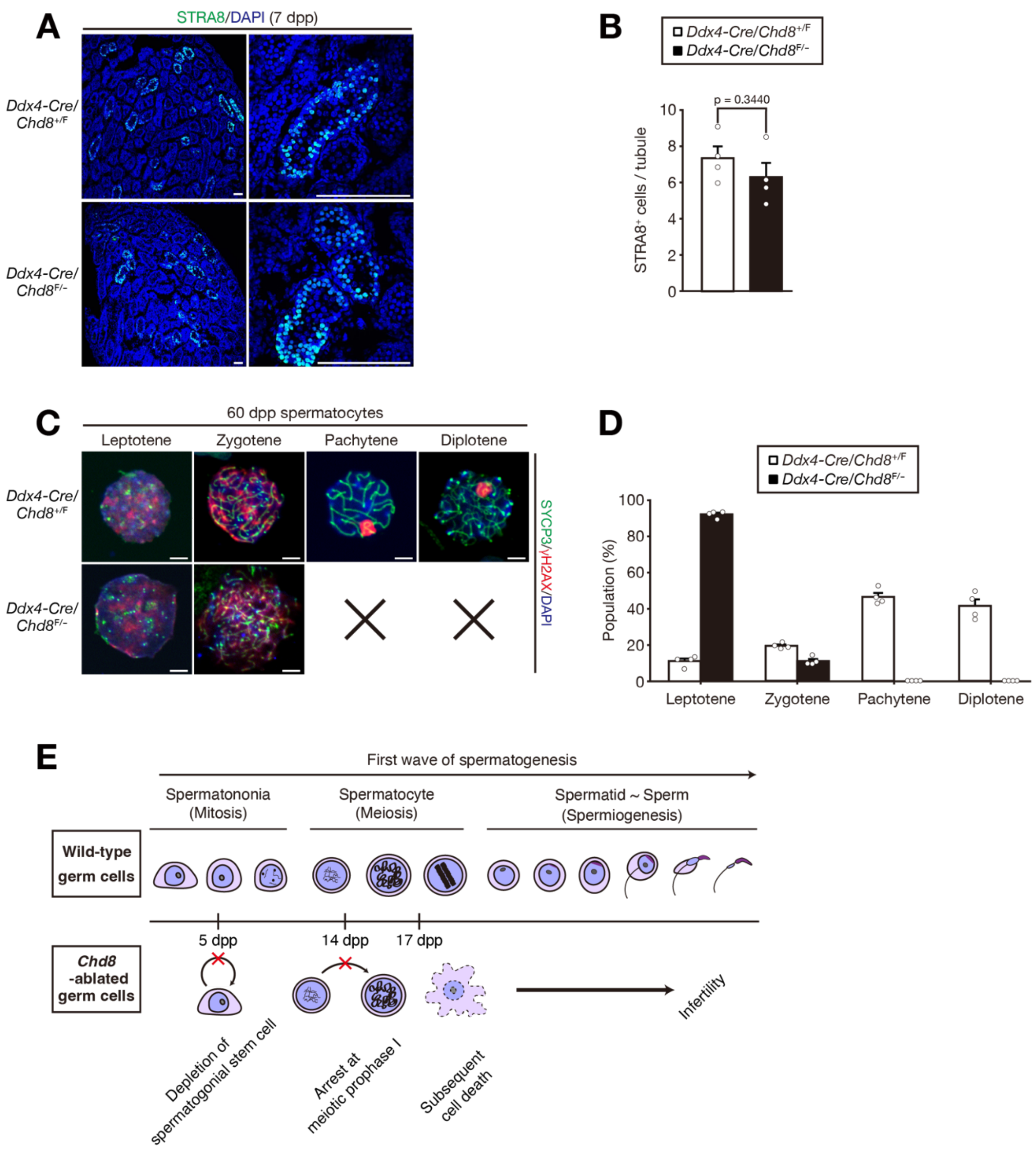

Figure S3. Loss of CHD8 leads to arrest before pachytene of meiotic prophase I and subsequent cell death

(A) Immunofluorescence staining of STRA8 in the testes from the mice of the indicated genotypes at 7 dpp. Scale bars, 100 μm.

(B) Quantification of the number of cells positive for STRA8 per tubule in images as in (A) (n = 4 mice per genotype). Data are means ± SEM. *P* values are calculated using the two-tailed Student's *t* test.

(C) Immunofluorescence staining of SYCP3 and γH2AX in the spermatocytes from the mice of the indicated genotypes at 60 dpp. × represents that there is no cell entry in the indicated

category. Scale bars, 5μm.

(D) Quantification of the number of cells in each indicated meiotic phase as in (C) (n = 4 mice per genotype).

(E) Schematic of postnatal development of germ cells during the first wave of spermatogenesis, comparing control and *Chd8*-ablated germ cells. Dpp, day post-partum.

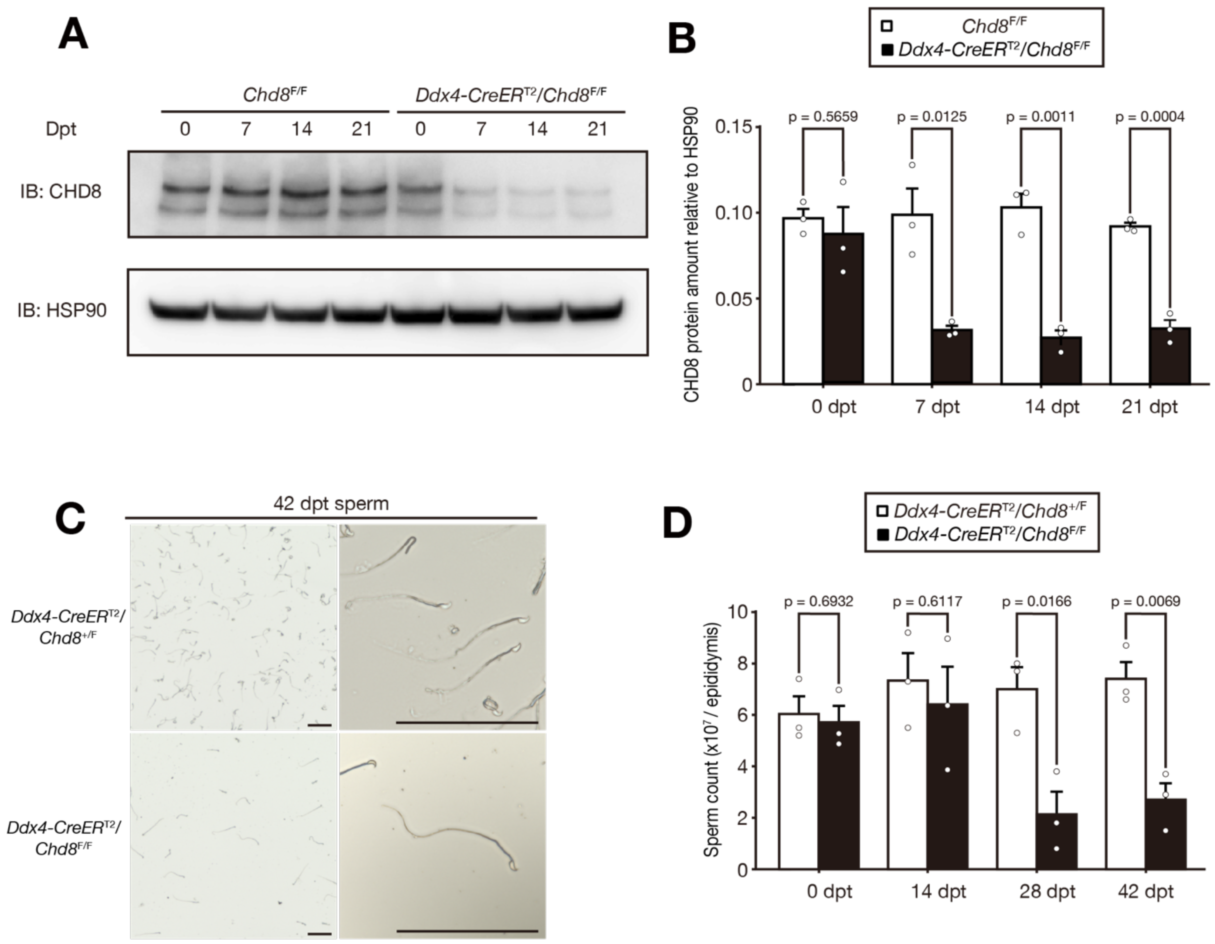

Figure S4. CHD8 is essential for the maintenance of adult spermatogenesis through two distinct functions

(A) Immunoblot analysis of CHD8 and HSP90 (loading control) was performed for the testes from the mice of the indicated genotypes at 0, 7, 14, and 21 dpt.

(B) Quantification of CHD8 abundance relative to HSP90 (loading control) in images as in (A) (n = 3 mice per genotype)

(C) Hematoxylin staining of mature sperm from the epididymides of the indicated genotypes at 42 days post-tamoxifen (dpt).

(D) Quantification of the number of mature sperm from the epididymides of the indicated genotypes at 7, 28, 60 dpp (n = 3 per genotype). All data are means ± SEM. *P* values are calculated using the two-tailed Student's *t* test.

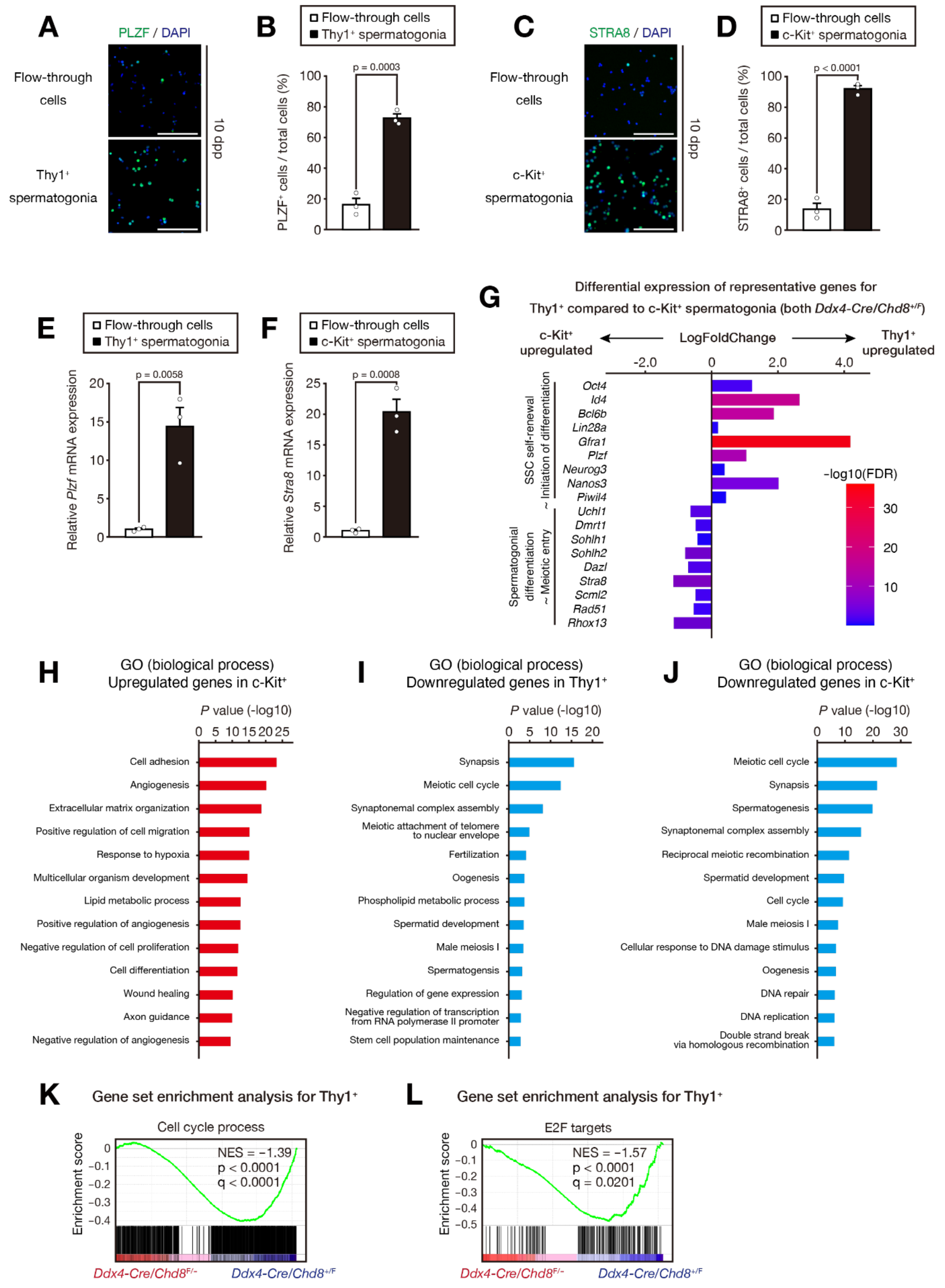

Figure S5. CHD8 is associated with the extensive activation of spermatogenic genes, including stem cell, spermatogonial differentiation, and meiosis-related genes

(A) Immunofluorescence staining of PLZF in testicular cells from the mice at 10 dpp. Scale bars, 100 μm.

(B) Quantification of the number of cells positive for PLZF as a percentage of the total number of cells in images as in (A) (n = 3 mice per genotype). Data are means ± SEM. *P* values are calculated using the two-tailed Student's *t* test.

(C) Immunofluorescence staining of STRA8 in testicular cells from the mice at 10 dpp. Scale bars, 100 μm.

(D) Quantification of cells positive for STRA8 as a percentage of the total number of cells in images as in (C) (n = 3 per cell-type). Data are means ± SEM. *P* values are calculated using the two-tailed Student's *t* test.

(E, F) RT-qPCR analysis of *Plzf* (E) and *Stra8* (F) mRNA in the cells of flow-through or MACS purified spermatogonia (n=3 per cell-type).

(G) Gene expression change of representative genes related to "SSC self-renewal ~ initiation of differentiation" and "Spermatogonial differentiation ~ meiotic entry" in Thy1+ spermatogonia compared with c-Kit+ spermatogonia. The comparison was performed within the spermatogonia of the same genotype, *Ddx4-Cre*; $Chd8^{+/F}$ (control group).

(H–J) Gene ontology analysis of up-regulated genes in $Thy1^{+}$ spermatogonia (H, 274 genes), down-regulated genes in $c\text{-}Kit^{+}$ spermatogonia (I, 2758 genes), and down-regulated genes in $c\text{-}Kit^{+}$ spermatogonia (J, 1377 genes) of *Ddx4-Cre*; $Chd8^{F/-}$ mice compared with *Ddx4-Cre*; $Chd8^{+/F}$ mice at 10 dpp (n = 3 per genotype). Dpp, day post-partum.

(K, L) GSEA plot of gene expression change among "Cell cycle process" genes (K) and "E2F targets" genes (L) in $Thy1^{+}$ spermatogonia.

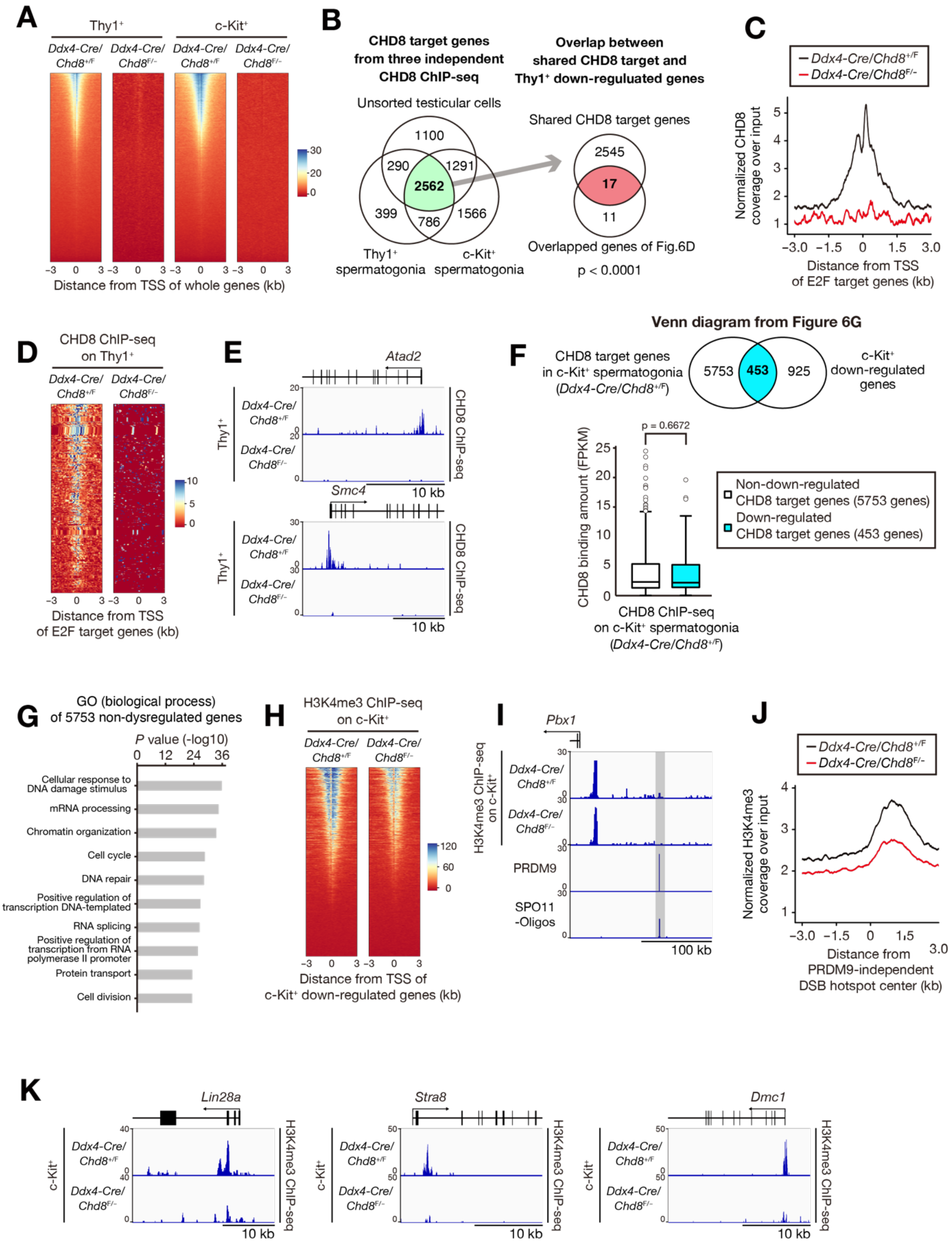

Figure S6. CHD8 transcriptionally regulates H3K4me3 histone methyltransferase, meiotic chromosome axis proteins, and DDR proteins to safeguard meiotic progression

(A) Heatmap clustering of CHD8 ChIP-seq peaks relative to TSSs of whole genes in Thy1⁺

and c-Kit$^+$ spermatogonia from the mice of the indicated genotypes.

(B) The Venn diagram on the left shows overlap among CHD8 target genes determined by three CHD8 ChIP-seq analyses in unsorted testicular cells of wild-type, Thy1$^+$ spermatogonia of *Ddx4-Cre*; *Chd8*$^{+/F}$, and c-Kit$^+$ spermatogonia of *Ddx4-Cre*; *Chd8*$^{+/F}$. The Venn diagram on the right shows overlap amongst the 2562 overlapping genes from the Venn diagram on the left and 28 genes from the Venn diagram in Figure 6D.

(C) Signal density of CHD8 peaks relative to TSSs of E2F target genes determined by ChIP-seq analyses of Thy1$^+$ spermatogonia from the mice of the indicated genotypes.

(D) Heatmap clustering of CHD8 ChIP-seq peaks relative to TSSs of E2F target genes in Thy1$^+$ spermatogonia from the mice of the indicated genotypes.

(E) CHD8 ChIP-seq data for representative E2F target genes in Thy1$^+$ spermatogonia from the mice of the indicated genotypes viewed in the Integrative Genomic Viewer browser.

(F) Box plot of CHD8 binding amount to TSSs, comparing 5753 non-down-regulated and 453 down-regulated genes in c-Kit$^+$ spermatogonia of *Ddx4-Cre*; *Chd8*$^{+/F}$ shown in the Venn diagram from Figure 6G.

(G) Gene ontology analysis of the 5753 CHD8 bound but non-down-regulated genes shown in (F).

(H) Heatmap clustering of H3K4me3 ChIP-seq peaks relative to TSSs of genes whose expression was down-regulated in c-Kit$^+$ spermatogonia from the mice of the indicated genotypes.

(I) H3K4me3 ChIP-seq data of the indicated genotypes along with PRDM9 ChIP-seq and SPO11-oligo data for representative DSB hotspots viewed in the Integrative Genomic Viewer browser. Shaded regions indicate DSB hotspots during meiotic homologous recombination. The data for PRDM9 ChIP-seq and SPO11-oligos were obtained from Grey *et al.* and Lange *et al.*, respectively.

(J) Signal density for H3K4me3 ChIP-seq peaks relative to PRDM9-independent DSB hotspot centers in c-Kit$^+$ spermatogonia from the mice of the indicated genotypes.

(K) H3K4me3 ChIP-seq data for representative spermatogenic genes in testicular cells from the mice of the indicated genotypes viewed in the Integrative Genomic Viewer browser. Dpp, days post-partum. DSB, double-strand break. FPKM, fragments per kilobase of transcript per million mapped reads. *P* values are calculated using "phyper" function in R (B) or Welch's t test (F).

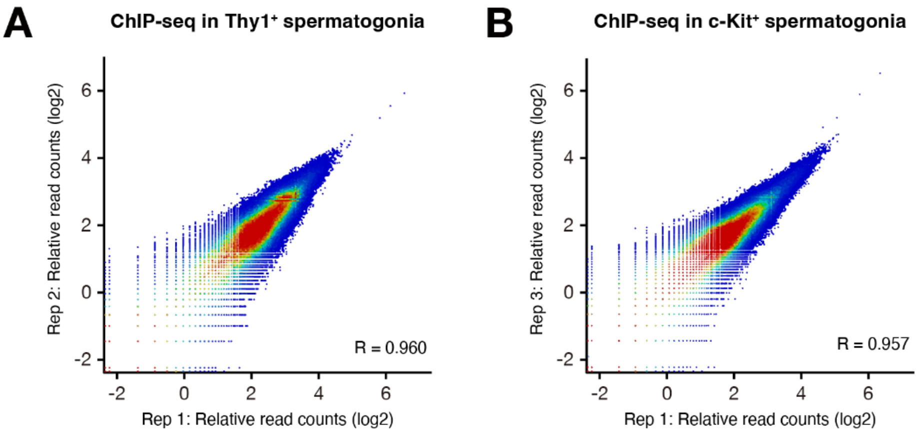

Figure S7. Biological replicates for CHD8 ChIP-seq data

(A, B) Scatter plot analyses comparing two independent biological replicates for CHD8 ChIP-seq profiles (bin = 10 kb) in (A) Thy1$^+$ and (B) c-Kit$^+$ spermatogonia at 10 dpp. R of each plot represents the Pearson correlation coefficient. Dpp, day post-partum.
